# The experience of providing end of life care at a children’s hospice: a qualitative study

**DOI:** 10.1186/s12904-017-0189-9

**Published:** 2017-02-13

**Authors:** Tracey McConnell, Sam Porter

**Affiliations:** 1 School of Nursing and Midwifery, Queen’s University Belfast, 97 Lisburn Road, Belfast, BT9 7BL United Kingdom; 2 Bournemouth University, Fern Barrow, Poole, Dorset BH12 5BB UK

## Abstract

**Background:**

More attention is being paid to the wellbeing of staff working in stressful situations. However, little is known about staff experience of providing end-of-life care to children within a hospice setting. This study aims to explore the experiences of care team staff who provide end-of-life care within a children’s hospice.

**Methods:**

Qualitative research incorporating interviews and a focus group. Data were analysed using thematic analysis. Purposeful sampling led to a total of 15 care team staff recruited from a children’s hospice offering palliative and specialist care to life-limited children and young people.

**Results:**

The hospice setting provides a model of excellence in supporting staff and mitigating challenging aspects of their role, which includes peer/organisational support, and regular ongoing training in key aspects of children’s palliative care. Key recommendations for improving their experience included advanced communication training and knowledge sharing with other children’s palliative care specialists within the acute setting.

**Conclusions:**

Service and policy initiatives should encourage open, informal peer/organisational support among the wider children’s palliative care sector. Further research should focus on paediatric palliative care education, particularly in relation to symptom management and communication at end-of-life, harnessing the expertise and breadth of knowledge that could be shared between children’s hospices and hospital settings.

**Electronic supplementary material:**

The online version of this article (doi:10.1186/s12904-017-0189-9) contains supplementary material, which is available to authorized users.

## Background

There is an increasing focus on examining the experiences and wellbeing of healthcare staff providing key services in order to improve patient/carer experience [1]. Staff who provide end-of-life care are more likely to experience high levels of emotional exhaustion [1] and as such require special attention.

Previous research suggests that staff who provide end-of-life care to children across a range of settings such as hospital and community, do report positive experiences, including rewarding relationships with children and their families [2–4], and making a difference in terms of reducing physical and psychosocial suffering [5]. However, a number of challenges have also been identified which may impact on staff wellbeing. These include stressful work environments, with shortage of staff and poor co-ordination between services cited as causing frustration and feelings of guilt over perceived inadequate care of the child and their family [6–8]. Making end-of-life decisions, symptom management [9, 10], and communicating with families about end-of-life care [6, 8] appear to be particularly challenging for staff. Many staff have also reported fears of expressing their own grief in public as they felt this would negatively impact the child’s family and their professional reputation [6, 8]. These challenging experiences have been found to have a direct correlation with staff wellbeing, and in turn patient/carer experience [11]. It is imperative that healthcare organisations examine their staff wellbeing regularly so they can trouble shoot areas of concern, and disseminate models of good practice [1].

Although the impact of providing end-of-life care to children is well documented [2, 6, 8], there is a dearth of literature in relation to staff who work within the hospice setting [12]. This study aims to address that gap by exploring staff experience in a children’s hospice setting, and how they cope with this unique role. While end-of-life care is often used synonymously with palliative care [1], for this study end-of-life, as understood by the staff taking part, is defined as the point when a child is considered to be actively dying.

### Aim

The aim of this study was to explore the impact of providing end-of-life care to children on staff within a hospice setting, how they cope, and draw recommendations for improving staff wellbeing, thereby improving the quality of paediatric care for both children and their families.

## Methods

This research took a qualitative approach to draw out the depth of staff’s experience. Individual interviews were used to explore in-depth experiences [13], and focus groups to identify group perspectives on the topic under investigation [14].

This study focused on the experience of the Care Team within a children’s hospice in Northern Ireland, who provide specialist palliative care services to children and their families. These include planned and emergency short breaks, symptom management, family support, end-of-life care, and bereavement support. Services are provided within the hospice facilities, as well as in the child’s own home. Care staff were recruited using a purposeful sampling approach from the total population of all care team staff working within the hospice (*n* = 40). Potential participants were contacted by administrative staff on the researcher’s behalf inviting them to take part in interviews, a focus group, or both if they wished. Information sheets for both interview and focus groups were attached, describing the purpose and nature of the research, along with assurances of confidentiality. The researcher’s details were provided with a request to get in contact if they wished to take part. A follow-up email was sent to non-responders after two weeks in order to aid recruitment.

Twelve semi-structured face-to-face interviews lasting between 30 to 45 min, and a focus group lasting approximately 60 min took place within a private room at the hospice at a date and time that was most convenient for the staff involved. Interviews were stopped at the point of data saturation. TM, an experienced female qualitative researcher with no prior relationship with participants conducted both interviews and the focus group with a range of consenting staff including nurses, healthcare assistants, and a care team manager. This hospice is a nurse-led unit, with sessional cover provided by GP’s on a daily basis. However, as medical input at end-of-life is intermittent, medical staff were not included. Data was collected during November 2015 to January 2016.

A semi-structured interview guide was developed based on previous work of the research team (see Additional file 1) [12]. The interview guide was structured around the research objectives, with prompts relating to key themes from this previous work [12]. A topic guide for the focus group was developed from key themes in the semi-structured interviews, along with prompts to draw out any issues participants felt were not adequately addressed in the literature (see Additional file 2). Both guides were pilot tested with senior staff at the hospice. Ethics approval was given by The School Research Ethics Committee at Queen’s University Belfast. All participants provided their written, informed consent.

### Analysis

Interviews and the focus group were audio recorded, transcribed verbatim by an independent transcriber, and checked for accuracy by T.M. Transcripts were analysed using a thematic analysis based on Newell and Burnard’s framework [15]. Key points highlighted by participants were arranged into themes and sub-themes by T.M. A random selection of the transcripts were examined by S.P., discussed and consensus sought to validate key themes and sub-themes. Data analysis was confirmed by two participants A realist approach as outlined by Porter [16] was used to enhance both rigour and the ‘confidence criterion’ (the level of confidence practitioners have that the findings presented accurately portray and explain the issues being addressed, and will inform their practice). This involved searching for deviant cases during data analysis, that is, those that did not fit patterns in the data. Transferability was enhanced by thick description of staff experiences within the hospice setting. A reflexive journal was used throughout data collection and analysis to record decisions and reasons for them in relation to the researcher’s interests in staff wellbeing.

## Results

Twelve individual interviews and one focus group with three participants were conducted, involving eight staff nurses, three senior staff nurses, two healthcare assistants, one senior healthcare assistant, and one care team manager. Their length of employment at the hospice ranged from two months to 15 years, with the majority of nurses having previously worked within the acute hospital setting.

### Rewarding experiences

It is important to point out that, despite the sadness of the circumstances that are inevitably part of working life in a children’s hospice, staff found many aspects of the work very rewarding.

#### Making a difference

Staff repeatedly spoke of ‘making a difference’ as one of the most positive experiences. Staff acknowledged that end-of-life situations were the most difficult things families could ever go through, and the thing that brought them the most satisfaction was being able to make that time somewhat easier, through supporting them, managing their child’s symptoms, and providing individualised care and activities.

*My comparison to obviously… working in the hospital, here, you have the time and it is one-to-one care, and that in itself is a special gift that a hospice can give that no other situation or working environment can do. (S14).*

*A lot of families would describe it as a big hug, like they feel arms like round them… (S8)*

#### We’re all in this together

Staff found great comfort in knowing that they were never alone, on a personal or professional level, in providing end-of-life care to a child:

*And then, the care that we have for one another, as a team, is a really positive experience I’ve had in here. (S6)*

*All the nurses on the team are fantastic and they’re so supportive. The entire management are very supportive as well… it’s about everybody knowing what stage the child’s at, and working and sharing experiences together for that child’s benefit. (S9)*

### Challenges

While staff identified a number of rewarding experiences, it was also clear that providing end-of-life care to children was associated with many challenging experiences, such as finding the right combination of medications to alleviate end-of-life care symptoms and reduce any distress for the child and family;

*I find that [symptom management] difficult because you’re aware that you don’t want the parents to have that lasting memory of their child having seizures or being in pain (S5)*

*When it’s challenging, like when it’s difficult to control pain or symptom management is difficult, that can be when it gets really stressful because you are just thinking, oh, I need to do more, you really just want to make sure, all the time, that we’re doing 100%, (S2)*

Communication with families;

*You’re almost afraid of saying the wrong thing, because that time is what families are going to remember, so you feel a lot of pressure to get it right for them. (S8)*

*Knowing what to say… like after the child has passed, because nothing you can say really is going to…benefit them… So that’s definitely I think the hardest. (4)*

Managing their own grief; and

*I think…if you didn’t grieve after a family…you couldn’t just bottle all that up. You couldn’t just keep building that up and not letting any of that out. (S1)*

Balancing complex respite care alongside end-of-life care.

*the stressful situations are whereby, you’ve all that going on in the mix as well as having children in for respite, and quite complex children, and you’ve got that family there, and they’re in an end-of-life situation, absolutely devastating.(S2)*

However, staff also reported a number of coping strategies and unique aspects of the hospice setting that helped mitigate the challenging aspects of their role. These findings indicate a number of recommendations for the hospice setting and the wider children’s end-of-life care sector.

### Recommendations

#### Self-care and building resilience

Staff talked about the importance of having an outlet outside of work in order to maintain energy for both their work and home life. Staff exercised self-care in a variety of ways such as focusing on the positives of their work, taking comfort from their religious faith, keeping busy with housework, reading, exercise, hobbies, practising mindfulness, or talking to their work colleagues. Most of these practices helped staff clear their heads and get into a better place psychologically or emotionally.

*I’ll go home and I’ll be in like super like tidy mode or like, like just need to do something in the house… like a distraction, to distract me, I like reading as well, and I’ll like read and like kind of use that to get myself into a different place. (S6)*

#### Peer support

One of the key areas of support for staff was being able to talk openly with their colleagues, which provided a safe place where they did not have to worry about not being understood or breaking confidentiality. Coming into work was seen as a positive because of the support they received.

*I have a couple of really good friends in here, and because they work here, it’s easy to sound off to somebody because you know it doesn’t go any further. Unless you’re working in this environment, you wouldn’t understand just how intense it can be. (S12)*

#### Organisational support – open doors and sticky buns

Another key source of support came from managers and the Senior Social Worker. Staff found this really helped in terms of releasing the pressure they sometimes felt.

*Even from a management point of view, they’re very involved on the floor in what’s going on. I mean, there’s always a chance to talk, and they definitely encourage that as well. (S2)*

Nursing staff also spoke of how beneficial their clinical supervision was for reflecting on end-of-life situations and receiving reassurance that they had done all they could for the child and family. However, some staff felt that formal debriefing did have a place for some people, especially care assistants who did not have clinical supervision. While some liked the idea of formal debriefing sessions, others did not like sharing in a formal group setting. Hospice leadership appeared to be proactively trying to find a middle ground by providing a safe but more informal space for all staff to share their experiences. For example, all staff mentioned ‘sticky bun days’. This simple act of getting together over a cup of tea and buns provided a safe place for staff to unwind, and was seen as a more relaxed and spontaneous response to a particular situation.

*At the start, we used to have debriefs all the time when somebody died, but it was very formal type of thing, sitting in a room with one of your managers and just talking about it, which a lot of people didn’t feel comfortable with. (S12)*

*The managers have…they’re kind of called sticky-bun sessions … just after there’s been maybe, a child that we’ve been caring for for a long time who has died or, just a difficult time, and we would come round here with a cup of tea and just talk about it, and even that in itself is just a bit of a safe space where you can kind of bounce off each other. (S5)*

#### Ongoing education and training in communication skills

Communication was mentioned often as an area requiring ongoing education, especially in relation to having difficult conversations.

*I think further development in communication is paramount for all staff, especially working in this area. …some people have gone to maybe communication days, but that’s been at a more senior level. (S3)*

While some staff felt role-play would be useful, others felt that learning by experience was more beneficial. Most staff referred to the wealth of knowledge that could be drawn from more experienced staff, including both healthcare assistants and nurses who had worked at the hospice for a long time.

*I think we get good training, mostly though cascaded down from, previous experience, and that experience is invaluable, of people that have obviously worked here for a long time. (S5)*

*I think the more experienced nurses do feel more like confident in what to say, and I know that the bereavement counsellor in here, all the nurses talk about her. She always says the right thing, and I think it is just through experience. (S4)*

#### Dissemination of specialist skills and experience

Staff also recognised the hospice as a leading organisation in this aspect of care, with great potential for sharing their experiences with other organisations who care for children with potentially life threatening illnesses, and also learning from their experiences.

*I do think that we have a lot to offer and that should be shared. I think…just bringing, again, professionals in and saying, “Hey, we’re the Hospice, our door is open – come and see us!” I just feel that they’re very insular, all the specialisms, and they should actually open themselves up to discussions, because they’ve a lot of experience to offer and learn from each other. (S14)*

## Discussion

The findings presented in this study provide unique insights into the experience of healthcare staff providing end-of-life care within a children’s hospice, and, as such, add to the paucity of studies conducted in this setting.

Such experiences highlight important implications for consideration for both service and policy level initiatives within the children’s’ palliative care sector, particularly areas of excellence that could be translated to other settings, and recommendations on how to improve staff experience.

In line with findings from both hospital and community settings, staff viewed their role as rewarding in terms of the meaningful relationships developed with children and their families [11]. However, unique to the hospice setting was the ability to provide one-to-one care which brought staff the most job satisfaction. Staff felt that the unique resources provided by the hospice setting, such as the hydro-pool and memory making activities provided them the opportunity to really make a difference for the child and family.

A number of challenges, similar to those in the hospital and community setting were identified, such as symptom management [10], and communication. A unique challenge identified in the hospice setting was related to coping with multiple end-of-life care situations, and balancing end-of-life care while providing care to children with complex health care needs. However, the key difference between the hospice environment and other paediatric palliative care settings was the supportive working environment which helped mitigate these challenging aspects of providing end-of-life care to children.

Our findings support previous research conclusions that the hospice setting provides the gold standard in terms of peer and organisational support to staff who provide end-of-life care to children [17]. This has key implications for improving patient care as research has shown that patient experience is improved when staff feel supported by their peers and organisation [1]. Therefore a key recommendation from this research would be to disseminate this model of peer and organisational support to other paediatric palliative care settings and services [1].

While formal debriefing has been recommended by previous research as a key strategy for helping support staff [12, 18], new findings from this study suggest that more informal support in the form of an ‘open door’ policy where staff are encouraged, and feel comfortable to talk to either management or more experienced staff, along with regular informal supportive meetings that promote commensality (eating and drinking together) is more beneficial. Research has shown that eating together promotes bonding among colleagues which may in turn make it easier to share experiences and gain support from others [19].

Regular training sessions were also offered, and staff were free to suggest any additional education or training needs. Staff also saw more experienced colleagues as invaluable for easing anxieties in relation to communicating with families about end-of-life issues. However, there was also consensus that advanced communication skills training should be available to all staff, not just more senior staff. Harnessing the expertise of experienced staff would be a cost-effective way to deliver this training.

Similarly to findings from other paediatric palliative care settings, psychological self-care was identified as an important coping strategy [20, 21]. Therefore, research exploring the most effective psychological interventions for promoting staff wellbeing is required. One promising emerging approach is mindfulness based supportive therapy [22] which may support healthcare practitioners self-care. This approach may also help improve patient care by helping staff really be there for their patients, while simultaneously helping staff manage the difficulties around professional boundaries inherent in providing end-of-life care to children and their families [23].

Another key finding highlighted by staff, and reiterated in the literature [24], was the importance of knowledge translation and sharing between the hospice setting and a range of paediatric palliative care settings in order for staff to benefit from each other’s expertise. Staff felt this would help ensure every child with palliative care needs can access hospice services if that is their choice, and that all children receive the best possible end-of-life care regardless of setting.

### Limitations and strengths

The findings consider a single children’s hospice, with a small sample size, limiting transferability to other settings. Nonetheless, many of the findings substantiate results from previous research in a range of settings. However, further research in different children’s hospices would be useful to triangulate our findings and assess the degree to which our recommendations would be helpful in other contexts. While being open to healthcare assistants, the sample was mostly comprised of nurses, which makes it less representative of all care staff. Healthcare assistants may have unique experiences which should be explored further in future studies. Nonetheless, this study adds to the limited understanding of staff experience of providing end-of-life care within a children’s hospice.

## Conclusion

With the increasing recognition of the key relationship between staff wellbeing and patient/carer reported experience, the impetus is on both service and policy level initiatives to improve staff experience. Findings suggest areas for dissemination of learning and good practice from the children’s hospice setting should include open, informal peer/organisational support, regular, ongoing research, education and training in symptom management and communication skills, drawing on the expertise of more experienced staff. Knowledge transfer should also be encouraged between children’s hospice and hospital settings to draw from others expertise, so that ultimately the best possible end-of-life care is available to children regardless of setting.

## Additional files

Additional file 1: Interview schedule. (DOC 41 kb)
Additional file 2: Interview schedule for focus group. (DOC 44 kb)
