# Supplementary material for: The experience of providing end of life care at a children’s hospice: a qualitative study
Source: BMC Palliat Care. 2017 Feb 13;16:15. doi: 10.1186/s12904-017-0189-9 (PMC5307784; doi:10.1186/s12904-017-0189-9)
Supplement: Additional file 1: — Interview schedule. (DOC 41 kb) [file 12904_2017_189_MOESM1_ESM.doc]

**Interview Schedule**

**Staff experience of providing end-of-life care at a children’s hospice.**

Before beginning, go over the pre-interview brief with participants reminding them of confidentiality unless the researcher suspects mal-practice or child protection issues, which have not been declared to the appropriate agencies.

**Introduction questions relating to the participant’s experience.**

1. To begin, could you describe any positive experiences of providing end-of-life care to children?

a. Any rewards (personal and/or professional)?

b. Major sources of job satisfaction?

2. Do you ever worry about ‘Professional Boundaries’ in relation to relationships with children and their families?

a. Becoming too close or over involved?

3. How do you feel about your work environment?

a. The hospice itself?

b. Organisational issues e.g. hierarchy, red tape, workload etc?

c. Infrastructure e.g. coordination of services in relation to referrals etc.?

d. Resources e.g. staffing levels, funding etc?

e. Do you feel supported/valued by management?

4. How would you describe communication with your colleagues?

a. Good, difficult etc.?

b. Within different professional groups e.g. nurses, doctors, management, allied health professionals etc.?

5. Is there any follow up in terms of bereavement support for families?

a. If yes, how do you feel about this aspect of care for you as a professional?

b. If no, how do you feel about the absence of this aspect of care for you as a professional?

6. What are your most challenging experiences providing end-of-life care to children?

a. Decision making e.g. who makes the decision that a child is end-of-life?

b. Deciding to withdraw treatment, food, fluids?

c.. Symptom management?

d. Talking to children about death and dying?

e. Communicating with parents that their child is end-of-life?

**Questions related to impact of their experiences.**

7. What impact do you feel your experiences of providing end-of-life care to children have on you?

a. Personally?

- Do you feel you grieve when a child dies?

- Emotional reactions e.g. crying, irritability, stress, anger (child’s death unfair), sleep problems, withdrawal etc.

- Difficulty moving past the child’s death?

- Impact on home life?

b. Professionally?

- Compromised ability to work?

- Helplessness e.g. witnessing child’s suffering, parent’s suffering/distress

- Sick leave?

- Desire to leave this area of work?

8. Do you feel you are allowed to openly grieve/ mourn the loss of a patient?

a. In front of family/friends?

b. In front of colleagues?

**Questions relating to coping strategies.**

9. What do you think helps you cope in your role providing end-of-life care to children?

a. Sense of humour?

b. Religious/spiritual beliefs?

c. Finding meaning e.g. making sense of child’s death.

d. Attending child’s funeral/ rituals to help bring closure e.g. writing a poem, lighting a candle?

e. Self-care e.g. meditation, regular exercise etc.

10. Do you ever distance yourself from children/families to protect yourself?

11. Are you aware of any support systems that are available to you e.g. via your workplace, other organisations?

a. Have you ever used them?

b. If „yes‟, were they helpful/unhelpful? Why?

**Questions relating to recommendations for practice.**

12. Are there any key recommendations you would make for improving practice?

1. Education and/or training?
2. Organisational support:

- Debriefing after child’s death
- Time out (15-30 minutes)
- Paid time off to attend child’s funeral

1. Support network for colleagues?
2. Any other services that would enhance support for staff?

**Drawing the interview to a close.**

13. As we are reaching the end of the interview, is there anything else that you feel would be important for me, or other people to know about your experience?

14. Are there any questions that I haven’t asked that you were expecting?

15. How have you found talking about your experience today?

**Reminder: Debriefing session available**
