# Supplementary material for: The experience of providing end of life care at a children’s hospice: a qualitative study
Source: BMC Palliat Care. 2017 Feb 13;16:15. doi: 10.1186/s12904-017-0189-9 (PMC5307784; doi:10.1186/s12904-017-0189-9)
Supplement: Additional file 2: — Interview schedule for focus group. (DOC 44 kb) [file 12904_2017_189_MOESM2_ESM.doc]

**Interview Schedule – Focus Group**

**Staff experience of providing end-of-life care at a children’s hospice.**

Before beginning, go over the pre-interview brief with participants reminding them of confidentiality unless the researcher suspects mal-practice or child protection issues, which have not been declared to the appropriate agencies.

**Introduction questions relating to the participant’s experience.**

1. To begin, could you tell me a little about your role here?
2. Nurse/care assistant etc.?
3. How long you’ve worked here?
4. Previous experience?
5. Why you came to work at the children’s hospice?
6. Could you describe any positive experiences of providing end-of-life care to children?
7. Any rewards (personal and/or professional)?
8. E.g. Making a difference/ Team work
9. Close relationships built up with children and families

3. Do you ever worry about ‘Professional Boundaries’ in relation to relationships with children and their families?

a. Becoming too close or over involved?

4. What are your most challenging experiences providing end-of-life care to children?

a. Symptom management?

b. Communication - saying the right thing?

c. withdrawing treatment

d. professional boundaries – getting too involved

e. Run of it - multiple end-of-life situations

f. Grief

**Questions relating to coping strategies.**

5. What do you think helps you cope in your role providing end-of-life care to children?

a. Self-care – Distraction (housework, reading)

b. faith,

c. exercise,

d. talking it out – peer support

e. attending funeral – finding closure

f. organisational support – open door and sticky buns

**Questions relating to recommendations for practice.**

6. Are there any key recommendations you would make for improving practice?

1. Education and/or training

- dealing with different faiths at end-of-life
- communication
- roleplay/guidance from more experienced staff

1. support for junior members of staff
2. raising awareness – funding, more referrals
3. Any other services that would enhance support for staff?

**Drawing the interview to a close.**

7. As we are reaching the end of the interview, is there anything else that you feel would be important for me, or other people to know about your experience?

8. Are there any questions that I haven’t asked that you were expecting?

9. How have you found talking about your experience today?

**Reminder: Debriefing session available**
